# Investigation of triple-negative breast cancer risk alleles in an International African-enriched cohort

**DOI:** 10.1038/s41598-021-88613-w

**Published:** 2021-04-29

**Authors:** Rachel Martini, Yalei Chen, Brittany D. Jenkins, Isra A. Elhussin, Esther Cheng, Syed A. Hoda, Paula S. Ginter, Jeffrey Hanover, Rozina B. Zeidan, Joseph K. Oppong, Ernest K. Adjei, Aisha Jibril, Dhananjay Chitale, Jessica M. Bensenhaver, Baffour Awuah, Mahteme Bekele, Engida Abebe, Ishmael Kyei, Frances S. Aitpillah, Michael O. Adinku, Kwasi Ankomah, Ernest B. Osei-Bonsu, Saul David Nathansan, LaToya Jackson, Evelyn Jiagge, Lindsay F. Petersen, Erica Proctor, Petros Nikolinakos, Kofi K. Gyan, Clayton Yates, Rick Kittles, Lisa A. Newman, Melissa B. Davis

**Affiliations:** 1 Department of Surgery, Weill Cornell Medicine, 420 E 70th Street, New York City, NY 10021 USA; 2 Department of Genetics, University of Georgia, Athens, GA USA; 3 Department of Public Health Sciences, Henry Ford Health System, Detroit, MI USA; 4 Center for Bioinformatics, Henry Ford Health System, Detroit, MI USA; 5 Department of Biology & Center for Cancer Research, Tuskegee University, Tuskegee, AL USA; 6 Department of Pathology and Laboratory Medicine, Weill Cornell Medicine, New York, NY USA; 7 Rutgers New Jersey Medical School, Newark, NJ USA; 8 Department of Surgery, Komfo Anokye Teaching Hospital, Kumasi, Ghana; 9 Department of Pathology, Komfo Anokye Teaching Hospital, Kumasi, Ghana; 10 Department of Pathology, St. Paul’s Hospital Millennium Medical College, Addis Ababa, Ethiopia; 11 Department of Pathology, Henry Ford Health System, Detroit, MI USA; 12 Department of Surgery, Henry Ford Health System, Detroit, MI USA; 13 Directorate of Oncology, Komfo Anokye Teaching Hospital, Kumasi, Ghana; 14 Department of Surgery, St. Paul’s Hospital Millennium Medical College, Addis Ababa, Ethiopia; 15 Department of Surgery, Kwame Nkrumah University of Science and Technology, Kumasi, Ghana; 16 Directorate of Radiology, Komfo Anokye Teaching Hospital, Kumasi, Ghana; 17 University Cancer and Blood Center, Athens, GA USA; 18 Department of Population Sciences, City of Hope Comprehensive Cancer Center, Duarte, CA USA

**Keywords:** Genetics, Cancer, Breast cancer, Cancer epidemiology, Cancer genetics, Risk factors, Genetics research

## Abstract

Large-scale efforts to identify breast cancer (BC) risk alleles have historically taken place among women of European ancestry. Recently, there are new efforts to verify if these alleles increase risk in African American (AA) women as well. We investigated the effect of previously reported AA breast cancer and triple-negative breast cancer (TNBC) risk alleles in our African-enriched International Center for the Study of Breast Cancer Subtypes (ICSBCS) cohort. Using case–control, case-series and race-nested approaches, we report that the Duffy-null allele (rs2814778) is associated with TNBC risk (OR = 3.814, *p* = 0.001), specifically among AA individuals, after adjusting for self-indicated race and west African ancestry (OR = 3.368, *p* = 0.007). We have also validated the protective effect of the minor allele of the *ANKLE1* missense variant rs2363956 among AA for TNBC (OR = 0.420, *p* = 0.005). Our results suggest that an ancestry-specific Duffy-null allele and differential prevalence of a polymorphic gene variant of *ANKLE1* may play a role in TNBC breast cancer outcomes. These findings present opportunities for therapeutic potential and future studies to address race-specific differences in TNBC risk and disease outcome.

## Introduction

Breast cancer (BC) is caused by a combination of dynamic influences, which are typically unique for each individual, but frequently may include underlying heritable genetic risks. Particularly, breast cancer patients who have early onset, or pre-menopausal incidence, typically are carriers of germline mutations in key cancer genes^1,2^. However, population studies have shown disparities in BC incidence and mortality among ethnic and racial groups persistently over the past five decades. In the US, White/European Americans (EA) have historically demonstrated the highest incidence of breast cancer, while Black or African Americans (AA) have the highest mortality rates reported in any race/ethnic group^3,4^. Interestingly, this mortality gap only emerged in the late 1970s, coinciding with implementation of targeted hormone therapies. The consequential decrease of mortality in EA was not matched in AA, which aside from unequal access to these new therapies, unmasked a race-group bias in breast tumor biology and incidence rates of tumor subtypes. Population studies of hormone receptor (HR) status in breast cancer diagnoses indicates a two-fold increased risk of Triple Negative Breast Cancer (TNBC) in AA compared to EA patients, which persists after adjusting for stage and age at diagnosis^5–8^. This trend also extends beyond certain social determinants, with AA having the highest rate of TNBC at every poverty level as well^9^. This finding translates to disproportionate survival benefits in EA patients from the standard-of-care targeted therapies that are primarily designed to target HRs^10^, which AA diagnosed with TNBC are not eligible to receive. Clinically, TNBC is a confirmed adverse prognostic feature in patients overall^11^, and in AA patients specifically^12^, and it underscores a need to identify any unique risk of certain breast cancer subtypes. An investigation of genetic risk across self-identified AA groups becomes more informative with the inclusion of an individual’s genetic ancestry composition, as levels of African versus European or other ancestry may be found at varying levels among this admixed population. For example, genetic risk in particular ancestral groups could be unmasked by investigating risk alleles within the predominant ancestral group, as opposed to the traditional risk studies that were devoid of ancestry data^13^. However, there is a severe shortage of genetic and GWAS data in non-white populations^13,14^, where less than 10–15% of individuals in population studies are Black, Indigenous, and People of Color (BIPOC), if race or ethnicity groups are reported at all^13^. This tragic limitation stifles our efforts to identify population-specific risk alleles outside of European descendant groups. However, recent studies have investigated race-specific risk; including, the Multi-Ethnic Cohort (MEC)^15^, the African American Breast Cancer Epidemiology and Risk (AMBER) Consortium^16–19^ (which includes the MEC), and our International Center for the Study of Breast Cancer Subtypes (ICSBCS), along with others^14,20–22^, are paving the way to more inclusion of AA and African participants in genomic research.

Previous studies inferred that AA-specific risk alleles held race-group specificity due to shared African genetic ancestry among AAs^15,23^. Through our Oncologic Anthropology epidemiological studies of breast cancer incidence and prevalence across the African Diaspora^24,25^, we have revealed a common trend of lower incidence but higher mortality among women of African descent^26^. Globally, there is also higher frequency of TNBC among women of western sub-Saharan African descent within every country that has a substantial population of individuals of African descent, and where we could investigate HR status, coupled with higher distribution of poor prognosis in these groups as well^7,27–30^. This strikingly correlates with the social history and unparalleled numbers of Africans dispersed during forced migrations of the Trans-Atlantic Slave trade, where over hundreds of years and a dozen generations, enslaved Africans were scattered across Europe, the Americas and the Caribbean.

We previously reported our independent analysis of AA race-group specific risk and our previous findings were able to replicate some, but not all, BC and TNBC-specific risk alleles in our African-enriched ICSBCS cohort^31^. Distinctions in risk associations from hazard models between cohorts could be confounded by bias in shared ancestry, due to differences in composition of genetic admixture among AAs. In this report, we reconsidered our previous risk findings to determine their relevance from a more global perspective, by (i) including additional ancestral populations from contemporary African women, and (ii) adjusting risk models for bias in ancestry background within admixed AAs. These efforts will provide further evidence and methodological insight in the role of shared African ancestry in the shared racial disparity of TNBC incidence across the African diaspora.

## Results

### Multi-ethnic cohort analysis of population-specific BC risk alleles reaffirms race group specific effects

**Figure 1:**
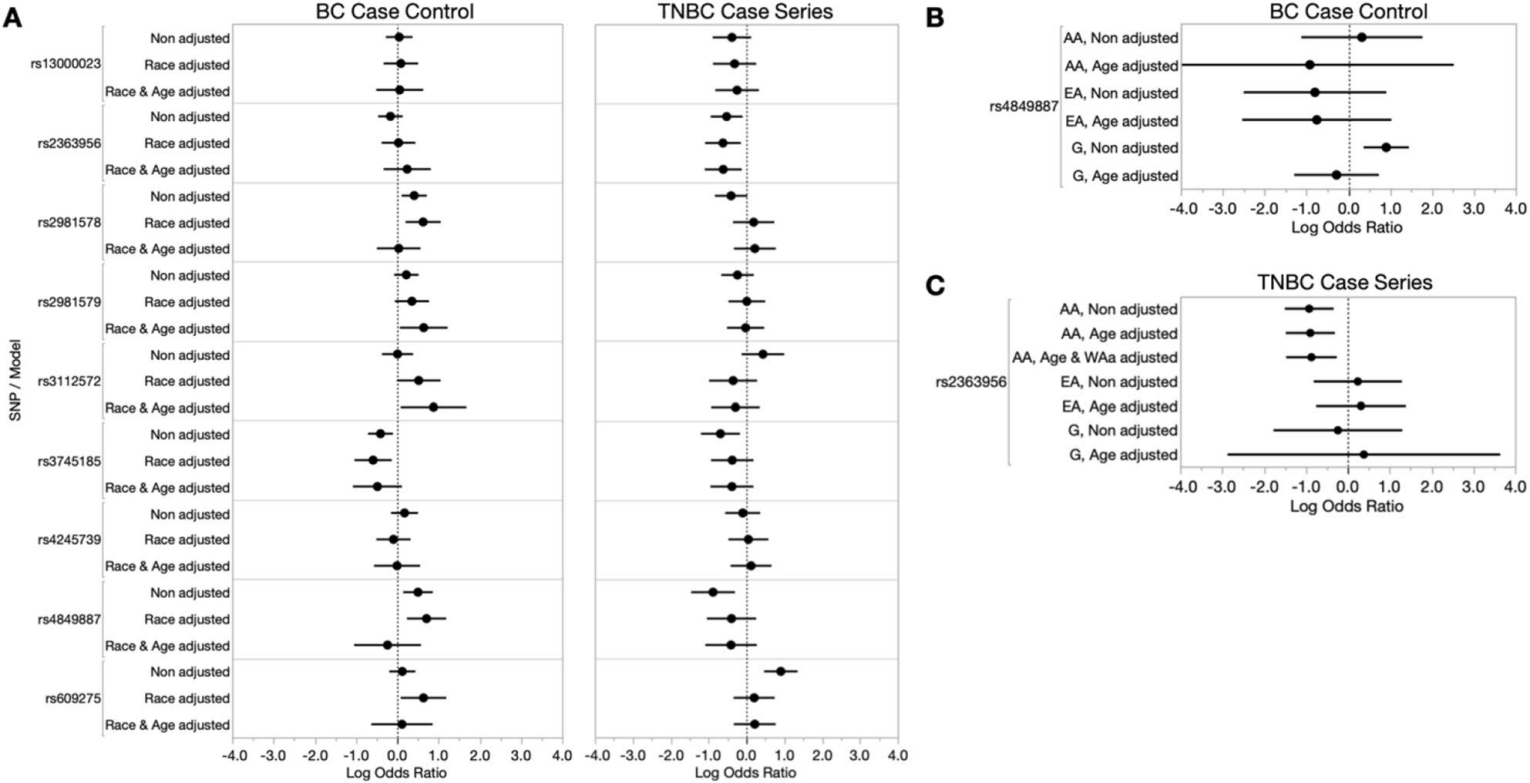
Breast cancer case–control and TNBC case-series risk analysis of previously identified BC risk alleles among our ICSBCS cohort. (**A**) The log odds ratio (x-axis) depicting SNV association with BC- or TNBC-risk among all samples is shown in non-adjusted models, and models adjusted for covariates (race and age) in our BC case–control analysis (left) and TNBC case-series analysis (right). (**B**) Within SIR BC case–control risk analysis for rs4849887. (**C**) Within SIR TNBC case-series analysis for rs2363956. For both (**B**) and (**C**), non-adjusted and age-adjusted models within SIR groups are shown for African Americans (AA), European Americans (EA), and Ghanaians (labelled as G). In our TNBC case-series analysis among SIR AA, we additionally adjusted for West African ancestry (WAa).

Our overall BC risk assessment model was an all-inclusive analysis, including all breast cancer subtypes and self-indicated race (SIR)/ancestral groups, where we have expanded the number of BC cases from Eastern and Western African nations, investigating previously published BC risk alleles that have been validated among African American women in the AMBER consortium^32^ (Tables 1, 2, Fig. 1A (left)). No strong linkage disequilibrium was observed among these alleles (maximum r^2^ of 0.44). Three alleles replicated previous associations of increased overall BC risk in our unadjusted models. These include rs2981578 (*FGFR2*), rs4849887 (*GLI2*), and rs3745185 (*BABAM1*). Interestingly, we found that the T allele of rs2981578 in the *FGFR2* gene was associated with increased risk (OR = 1.508, *p* = 0.008491), which contrasts with previous reports of the C allele as the risk allele. The C allele of rs4849887 in the *GLI2* gene was associated with increased risk (OR = 1.654, *p* = 0.006122), replicating previous findings. We also replicated the protective A allele of rs3745185 in the *BABAM1* gene (OR = 0.67, *p* = 0.008402).

**Table 1:** Population frequencies of candidate alleles for BC and TNBC-specific risk analyses.

SNP	Associated/neighboring gene	Chr:position	Variant consequence	Minor allele	ICSBCS Cohort*				1000 Genomes**		
					AA MAF	EA MAF	G MAF	E MAF	Global MAF	AFR MAF	EUR MAF
rs13000023	*TNP1, DIRC3*	2:217,924,394	Intron	A	0.219	0.268	0.244	0.155	0.150	0.130	0.210
rs2363956	*ANKLE1*	19:17,394,124	Missense	G	0.482	0.510	0.490	0.683	0.460	0.500	0.570
rs2981578	*FGFR2*	10:123,340,311	Intron	T	0.271	0.516	0.364	0.250	0.370	0.080	0.480
rs2981579	*FGFR2*	10:123,337,335	Intron	G	0.398	0.551	0.449	0.440	0.510	0.340	0.550
rs3112572	*CASC16, LOC643714*	16:52,600,447	Intron	A	0.216	0.011	0.246	0.207	0.120	0.270	0.030
rs3745185	*BABAM1*	19:17,384,267	Intron	A	0.191	0.400	0.261	0.369	0.310	0.210	0.480
rs4245739	*MDM4*	1:204,518,842	3′ UTR	C	0.271	0.321	0.252	0.145	0.210	0.230	0.260
rs4849887	*LOC84934, GLI2*	2:121,245,122	Intergenic	C	0.349	0.495	0.329	0.440	0.790	0.700	0.900
rs609275	*MYEOV, CCND1*	11:69,402,915	Regulatory	T	0.491	0.037	0.552	0.085	0.210	0.490	0.000
rs6676002	*DARC/ACKR1*	1:159,173,144	Upstream	T	0.027	0.212	0.000	0.195	0.090	0.010	0.180
rs3027008	*DARC/ACKR1*	1:159,173,539	Upstream	T	0.027	0.212	0.012	0.195	0.090	0.010	0.180
rs3027013	*DARC/ACKR1*	1:159,174,209	5′ UTR	T	0.022	0.087	0.000	0.110	0.030	0.000	0.080
rs71782098	*DARC/ACKR1*	1:159,174,347	5′ UTR	DEL	0.080	0.021	0.103	0.060	0.030	0.100	0.000
rs2814778	*DARC/ACKR1*	1:159,174,683	5′ UTR	C	0.797	0.005	0.971	0.533	0.270	0.960	0.010
rs17838198	*DARC/ACKR1*	1:159,175,005	Intron	T	0.084	0.263	0.005	0.131	0.220	0.010	0.230
rs3027016	*DARC/ACKR1*	1:159,175,193	Splice/Intron	G	0.045	0.163	0.000	0.037	0.060	0.000	0.160
rs12075	*DARC/ACKR1*	1:159,175,354	Missense	G	0.128	0.398	0.012	0.207	0.460	0.020	0.400

*African Americans (AA), European Americans (EA), Ghanaians (G), Ethiopians (E).

*MAF = Minor allele frequency.

**1000 Genomes Allele frequencies obtained from ensemble.

**Table 2:** Breast cancer risk assessment (case–control) of previously identified variant alleles.

SNV ID	Associated/neighboring genes	Minor allele	Overall BC risk (all samples)						SIR African Americans			SIR Ghanaians		
			Models without covariates			Models with covariates*			Models with covariates**			Models with covariates**		
			*N*	OR (95% CI)	*P *value	*N*	OR (95% CI)	*P* value	*N*	OR (95% CI)	*P* value	*N*	OR (95% CI)	*P* value
rs13000023	*TNP1, DIRC3*	A	420	1.048 (0.756, 1.449)	0.775	271	1.061 (0.601, 1.874)	0.838	104	–	–	75	0.909 (0.466, 1.770)	0.780
rs2363956	*ANKLE1*	G	415	0.847 (0.629, 1.140)	0.273	274	1.272 (0.718, 2.254)	0.409	103	0.916 (0.135, 6.197)	0.928	76	1.409 (0.688, 2.890)	0.349
**rs2981578**	***FGFR2***	T	416	***1.508 (1.111, 2.047)***	***0.008***	267	1.036 (0.611, 1.759)	0.895	99	–	–	78	0.891 (0.481, 1.650)	0.713
**rs2981579**	***FGFR2***	G	418	1.246 (0.928, 1.675)	0.144	269	***1.899 (1.063, 3.393)***	***0.030***	99	–	–	77	1.792 (0.905, 3.545)	0.095
**rs3112572**	***CASC16, LOC643714***	A	385	1.007 (0.691, 1.467)	0.972	246	***2.410 (1.086, 5.347)***	***0.031***	96	–	–	65	2.088 (0.948, 4.597)	0.068
**rs3745185**	***BABAM1***	A	409	***0.666 (0.492, 0.901)***	***0.008***	265	0.618 (0.341, 1.119)	0.112	99	0.787 (0.104, 5.947)	0.816	77	0.590 (0.272, 1.280)	0.182
rs4245739	*MDM4*	C	409	1.193 (0.860, 1.654)	0.291	264	0.995 (0.569, 1.740)	0.986	97	0.008 (0.000, 20.16)	0.228	75	0.838 (0.427, 1.644)	0.607
**rs4849887**	***LOC84934, GLI2***	C	403	***1.654 (1.154, 2.371)***	***0.006***	257	0.790 (0.351, 1.776)	0.568	100	0.399 (0.013, 12.43)	0.601	69	0.754 (0.274, 2.073)	0.584
rs609275	*MYEOV, CCND1*	T	396	1.129 (0.822, 1.552)	0.453	253	1.121 (0.532, 2.361)	0.764	98	0.224 (0.014, 3.646)	0.293	67	0.920 (0.389, 2.176)	0.849

Significant alleles (*p* value < 0.05) are given in bold and italic

*Overall analysis models with covariates adjusts for age and SIR.

**SIR models with covariates adjusts for age.

To determine whether these all-inclusive association models may be confounded by race-specific bias in age or allele frequency, we adjusted the risk model to correct for race and age. Interestingly, each unadjusted risk association loses significance in the combined race group model after adjusting for race and age, indicating that the risk alleles may have higher frequency in one of the SIR groups (See Table 1). Specifically, in the case of the risk (C) allele of rs4849887, we find it is 10–15% lower in populations of West African descent (AA = 34.9%, Ghanaians = 32.9%), compared to European Americans (49.5%) and East Africans (44.0%) in our cohort. Two additional alleles gained significance in overall BC risk associations after race and age adjustments in our all-inclusive model, rs2981579 in the *FGFR2* gene (OR = 1.899, *p* = 0.03038) and rs3112572 in the *LOC643714* gene (OR = 2.410, *p* = 0.03055).

Next, we tested whether the associated BC risk of our candidate alleles was different among SIR groups by performing a nested BC risk assessment within each of the SIR groups (Table 2 and Supplemental Table 1). While we observed rs4849887 was associated with overall BC risk prior to adjusting for age and race, this allele is associated with higher overall BC risk only in Ghanaians prior to adjusting for age (OR = 2.472, *p* = 0.001032) (Fig. 1B, Supplemental Table 1). While we did not observe a significant association between rs609275 and overall BC risk for the whole cohort assessment, a very high overall BC risk was observed specifically for AA prior to adjusting for age (OR = 5.383, *p* = 0.048). There were no significant associations found between the previously identified variants and breast cancer risk among SIR EA in both unadjusted and age-adjusted models (Supplemental Table 1).

### TNBC-specific case-series analysis of population-specific BC risk alleles shows associations within ancestral groups

The higher rate of TNBC among women of African descent worldwide begs the question of whether there is a shared genetic risk among the African diaspora, and we have previously shown that quantified West African ancestry was strongly associated with TNBC disease^31^*.* Using a case-series analysis in our African-enriched cohort, we tested whether previously reported AA-specific risk alleles were associated specifically with TNBC disease risk (Table 3, Supplemental Table 2, Fig. 1A (right)). Prior to adjusted covariate modeling, five of the nine AA-risk variants showed significant association with TNBC disease risk. Four of these variants were not previously reported as having ER-negative disease specific risk, and four were predicted to have a protective effect; including, rs2981578 in *FGFR2* (OR = 0.667, *p* = 0.0627), rs3745185 in *BABAM1* (OR = 0.503, *p* = 0.009), rs4849887 in *GLI2 (*OR = 0.414, *p* = 0.003), and rs2363956 in *ANKLE1* (OR = 0.593, *p* = 0.0149). Only the SNV rs609275 in *MYEOV/CCND1* showed higher hazard/risk for TNBC in the unadjusted model (OR = 2.479, p = 5.68E-05). The *ANKLE1* variant rs2363956 replicated in the TNBC/ER-negative specific protective effect that was previously reported and was the only variant to retain significance after adjusting for race and age (OR = 0.542, *p* = 0.014).

**Table 3:** TNBC-specific risk assessment (case-series) of previously identified variant alleles.

SNV ID	Associated/neighboring genes	Minor allele	Overall TNBC risk (all samples)						SIR African Americans			SIR Ghanaians		
			Models without covariates			Models with covariates*			Models with covariates**			Models with covariates***		
			*N*	OR (95% CI)	*P *value	*N*	OR (95% CI)	*P* value	*N*	OR (95% CI)	*P* value	*N*	OR (95% CI)	*P* value
rs13000023	*TNP1, DIRC3*	A	197	0.682 (0.410, 1.133)	0.139	190	0.781 (0.439, 1.387)	0.399	96	1.109 (0.538, 2.286)	0.780	6	–	–
**rs2363956**	***ANKLE1***	G	201	***0.593 (0.389, 0.903)***	***0.015***	194	***0.542 (0.332, 0.883)***	***0.014***	95	***0.420 (0.230, 0.769)***	***0.005***	8	1.471 (0.057, 38.26)	0.816
rs2981578	*FGFR2*	T	190	0.667 (0.435, 1.022)	0.063	183	1.248 (0.718, 2.169)	0.432	92	1.304 (0.656, 2.590)	0.449	6	17.55 (0.010, 29,760)	0.450
rs2981579	*FGFR2*	G	193	0.790 (0.515, 1.212)	0.280	186	0.978 (0.599, 1.597)	0.930	92	0.942 (0.530, 1.672)	0.838	6	6.261E−08 (0, –)	0.993
rs3112572	*CASC16, LOC643714*	A	181	1.546 (0.880, 2.716)	0.130	174	0.748 (0.394, 1.421)	0.375	89	0.714 (0.367, 1.390)	0.322	5	6.976E−49 (0, –)	0.976
**rs3745185**	***BABAM1***	A	189	***0.503 (0.306, 0.843)***	***0.009***	182	0.682 (0.386, 1.203)	0.180	92	0.584 (0.288, 1.182)	0.135	6	3.304 (0.090, 120.90)	0.515
rs4245739	*MDM4*	C	190	0.906 (0.571, 1.438)	0.675	183	1.130 (0.658, 1.941)	0.657	90	0.861 (0.440, 1.685)	0.663	6	0.356 (0.004, 34.530)	0.658
**rs4849887**	***LOC84934, GLI2***	C	189	***0.414 (0.232, 0.738)***	***0.003***	182	0.666 (0.338, 1.313)	0.240	93	0.542 (0.241, 1.221)	0.140	6	0.676 (0.0470, 9.710)	0.773
**rs609275**	***MYEOV, CCND1***	T	187	***2.479 (1.593, 3.857)***	** < 0.001**	180	1.245 (0.717, 2.163)	0.437	91	1.121 (0.610, 2.061)	0.714	6	14.23 (0.0261, 7750)	0.409

Significant alleles (*p* value < 0.05) are given in bold and italic

*Overall analysis models with covariates adjusts for age and SIR.

**SIR AA models with covariates adjusts for age and West African ancestry.

***SIR Ghanaian models with covariates adjusts for age.

Similar to our BC case–control analysis, we used a nested risk analysis within SIR groups to test for SIR-specific risk. For the admixed AA population, we included quantified West African ancestry (WAa) in the adjusted covariate modeling. The rs2363956 variant in the *ANKLE1* gene retained a protective effect for TNBC in AAs, even after covariate adjustments, (age and WAa adjusted OR = 0.4204, *p* = 0.005), indicating this is not a mere artifact of disequilibrium, or biased distribution of the allele in African populations (Fig. 1C and Table 3).

### DARC/ACKR1 alleles in BC and TNBC risk

**Figure 2:**
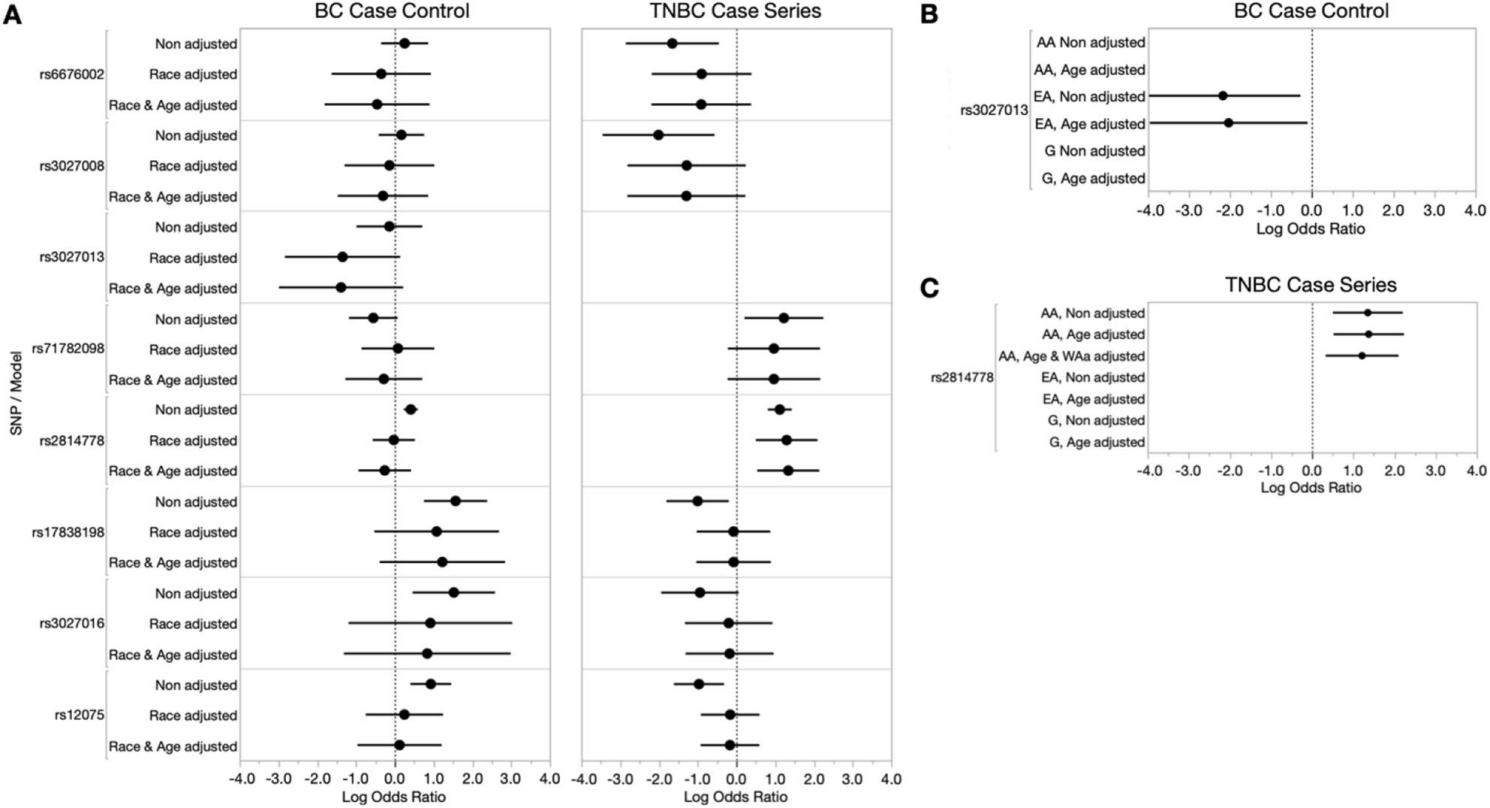
Breast cancer case–control and TNBC case-series risk analysis of *DARC/ACKR1* alleles among our ICSBCS cohort. (**A**) The log odds ratio (x-axis) depicting SNV association with BC- or TNBC-risk among all samples is shown in non-adjusted models, and models adjusted for covariates (race and age) in our BC case–control analysis (left) and TNBC case-series analysis (right). (**B**) Within SIR BC case–control analysis for rs3027013. (**C**) Within SIR TNBC case-series analysis for rs2814778. For both (**B**) and (**C**), non-adjusted and age-adjusted models within SIR groups are shown for African Americans (AA), European Americans (EA), and Ghanaians (labelled as G). In our TNBC case-series analysis among SIR AA, we additionally adjusted for West African ancestry (WAa).

In addition to the previously implicated AA-risk alleles, we have also included *DARC/ACKR1* alleles, including the TNBC risk associated Duffy-null allele^31^, to investigate whether alternative variants may capture risk due to unique biological contributions of either isoforms or distinct gene regulation (Table 1). Our new analysis found that four *DARC/ACKR1* SNVs also had significant potential to confer overall BC risk in our all-inclusive analysis models (rs2814778 OR = 1.512, *p* < 0.001, rs17838198, OR = 4.798, *p* < 0.001, rs3027016 OR = 4.586, *p* = 0.005 and rs12075 OR = 2.534, *p* < 0.001, respectively), however, after adjusting for age and race, this is mostly lost (Table 4, Fig. 2A (left)). In our SIR nested analysis model, the *DARC/ACKR1* variant rs3027013 showed a significant protective effect in EA patients, even after age-adjusted modeling (age-adjusted OR = 0.131, *p* = 0.03897) (Fig. 2B and Supplemental Table 3).

**Table 4:** Breast cancer risk assessment (case–control) of *DARC/ACKR1* alleles.

SNV ID	Associated/neighboring genes	Minor allele	Overall BC risk (all samples)						SIR African Americans			SIR Ghanaians		
			Models without covariates			Models with covariates*			Models with covariates**			Models with covariates**		
			*N*	OR (95% CI)	*P *value	*N*	OR (95% CI)	*P* value	*N*	OR (95% CI)	*P* value	*N*	OR (95% CI)	*P* value
rs6676002	*DARC/ACKR1*	T	282	1.290 (0.702, 2.368)	0.412	231	0.634 (0.164, 2.454)	0.509	96	–	–	47	–	–
rs3027008	*DARC/ACKR1*	T	282	1.187 (0.661, 2.131)	0.566	231	0.737 (0.230, 2.369)	0.609	96	–	–	49	2.270 (0.277, 18.60)	0.445
rs3027013	*DARC/ACKR1*	T	283	0.869 (0.372, 2.033)	0.747	231	0.250 (0.050, 1.241)	0.090	96	–	–	49	–	–
rs71782098	*DARC/ACKR1*	DEL	291	0.575 (0.307, 1.077)	0.084	238	0.753 (0.280, 2.028)	0.575	96	–	–	52	0.882 (0.298, 2.608)	0.820
**rs2814778**	***DARC/ACKR1***	C	712	***1.512 (1.263, 1.809)***	**< 0.001**	492	0.772 (0.392, 1.520)	0.454	153	0.696 (0.330, 1.467)	0.340	54	3.657 (0.231, 57.81)	0.357
**rs17838198**	***DARC/ACKR1***	T	299	***4.798 (2.125, 10.83)***	**< 0.001**	244	3.413 (0.678, 17.20)	0.137	97	1.052 (0.044, 25.41)	0.975	58	–	–
**rs3027016**	***DARC/ACKR1***	G	281	***4.586 (1.587, 13.26)***	***0.005***	229	2.311 (0.269, 19.88)	0.446	96	–	–	47	–	–
**rs12075**	***DARC/ACKR1***	G	292	***2.534 (1.498, 4.287)***	**< 0.001**	238	1.131 (0.382, 3.351)	0.824	97	1.108 (0.048, 25.80)	0.949	53	–	–

Significant alleles (*p* value < 0.05) are given in bold and italic

*Overall analysis models with covariates adjusts for age and SIR.

**SIR models with covariates adjusts for age.

For *DARC/ACKR1* variant associations in TNBC-specific risk, we similarly observed that seven out of eight variants were associated with TNBC disease, in which five of the minor alleles presented a protective effect and two showed increased risk, prior to race/age adjustments (rs6676002, OR = 0.191, *p* = 0.007; rs3027008, OR = 0.134, *p* = 0.006; rs17838198, OR = 0.367, *p* = 0.015; rs3027016, OR = 0.390, *p* = 0.065; rs12075, OR = 0.380, *p* = 0.003, rs71782098, OR = 3.403, *p* = 0.018; and rs2814778, OR = 3.062, *p* < 0.001) (Table 5, Fig. 2A (right)). Interestingly, as we previously reported with only AA and EA, the Duffy-Null allele, rs2814778, retained significant TNBC-risk association with the addition of West African samples, even after age and SIR adjustments (OR = 3.814, *p* = 0.001). The Duffy-Null (rs2814778) TNBC-risk association was also retained in our nested SIR analysis among AA, following both age and quantified West African ancestry adjustment (OR = 3.368, *p* = 0.007) (Fig. 2C and Table 5). This indicates that the TNBC-specific risk conferred by the Duffy-null allele in the *DARC/ACKR1* gene is not an artifact of shared ancestry bias, but rather an ancestry-specific risk allele.

**Table 5:** TNBC-specific risk assessment (case-series) of *DARC/ACKR1* alleles.

SNV ID	Associated/neighboring genes	Minor Allele	Overall TNBC risk (all samples)						SIR African Americans			SIR Ghanaians		
			Models without covariates			Models with covariates*			Models with covariates** Models with covariates**			Models with covariates***		
			*N*	OR (95% CI)	*P *value	*N*	OR (95% CI)	*P* value	*N*	OR (95% CI)	*P* value	*N*	OR (95% CI)	*P* value
**rs6676002**	***DARC/ACKR1***	T	176	***0.191 (0.058, 0.635)***	***0.007***	175	0.403 (0.111, 1.460)	0.167	90	–	–	2	–	–
**rs3027008**	***DARC/ACKR1***	T	174	***0.134 (0.032, 0.568)***	***0.006***	173	0.275 (0.060, 1.261)	0.097	90	–	–	2	–	–
rs3027013	*DARC/ACKR1*	T	174	–	–	173	–	–	90	–	–	2	–	–
**rs71782098**	***DARC/ACKR1***	DEL	178	***3.403 (1.231, 9.412)***	***0.018***	177	2.629 (0.796, 8.682)	0.112	90	2.547 (0.668, 9.716)	0.171	2	–	–
**rs2814778**	***DARC/ACKR1***	C	339	***3.062 (2.249, 4.168)***	**< 0.001**	304	***3.814 (1.710, 8.493)***	***0.001***	95	***3.368 (1.390, 8.165)***	***0.007***	16	–	–
**rs17838198**	***DARC/ACKR1***	T	178	***0.367 (0.164, 0.821)***	***0.015***	177	0.929 (0.355, 2.430)	0.881	91	0.722 (0.190, 2.754)	0.634	2	–	–
rs3027016	*DARC/ACKR1*	G	174	0.390 (0.144, 1.058)	0.065	173	0.839 (0.270, 2.609)	0.762	90	1.204 (0.226, 6.422)	0.828	2	–	–
**rs12075**	***DARC/ACKR1***	G	177	***0.380 (0.199, 0.726)***	***0.003***	176	0.846 (0.396, 1.807)	0.666	91	0.922 (0.319, 2.669)	0.881	2	–	–

Significant alleles (*p* value < 0.05) are given in bold and italic

*Overall analysis models with covariates adjusts for age and SIR.

**SIR AA models with covariates adjusts for age and West African ancestry.

***SIR Ghanaian models with covariates adjusts for age.

### Functional consequences of the TNBC-protective rs2362956 variant in ANKLE1

**Figure 3:**
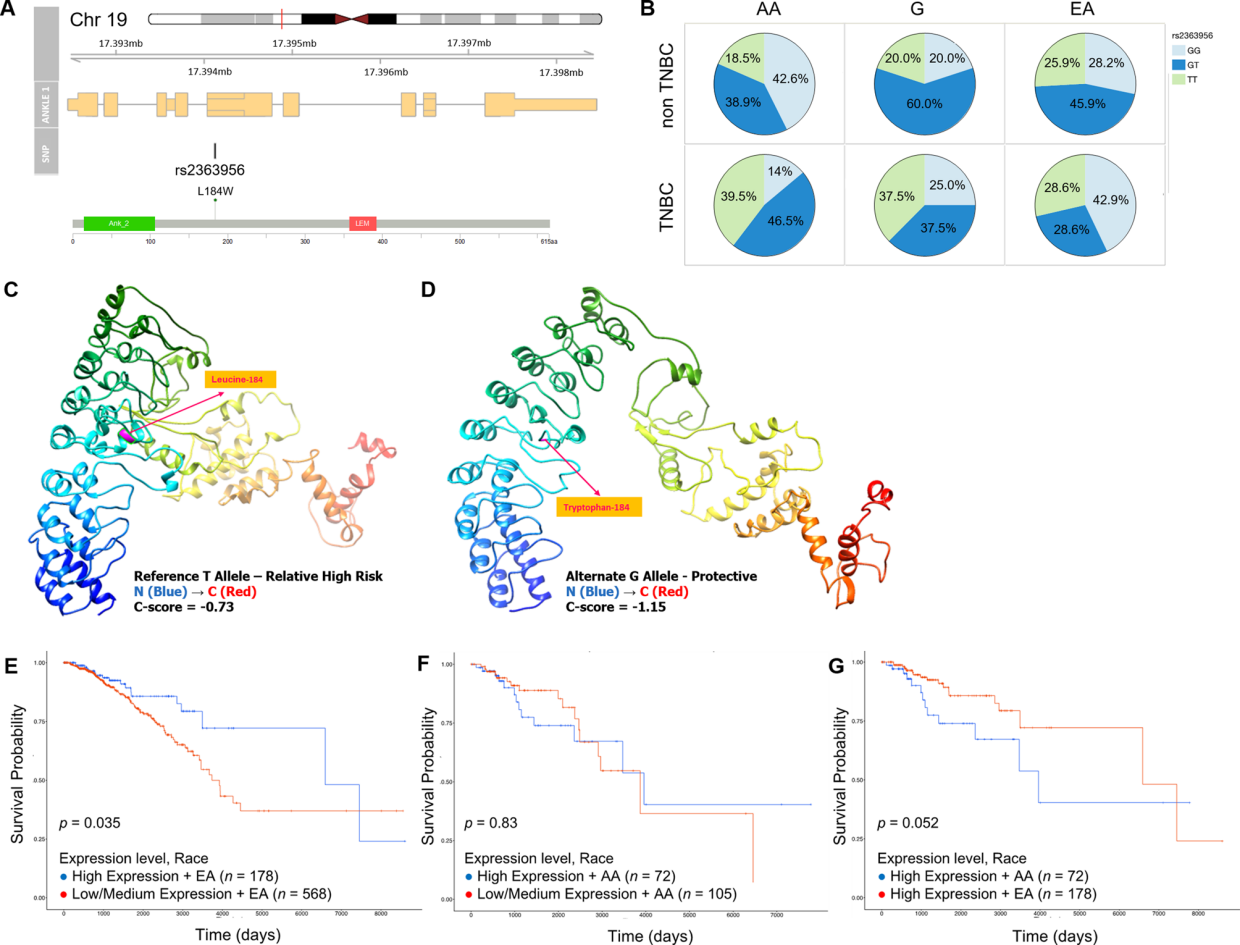
Functional implications of the *ANKLE1* variant rs2363956. (**A**) rs2363956 is a coding region variant of the *ANKLE1* gene, located at 19p13.11. This missense variant encodes a leucine to tryptophan change at amino acid position 184 (ANKLE1 protein domain model shown from cBioPortal^61^). (**B**) Genotype frequency pie charts of the rs2363956 allele among SIR African Americans (AA), SIR Ghanaian (G) and SIR European American (EA) individuals. Non-TNBC cases are shown in the top row, and TNBC cases are in the bottom row. Those individuals homozygous for the protective/minor G allele are shown in light blue, heterozygotes are dark blue, and individuals homozygous for the major T allele are in light green. (**C**) Illustration of the predicted 3D ANKLE1 protein structure from I-TASSER using Chimera with leucine at position 184 (representing the reference allele), and (**D**) with tryptophan at position 184 (representing the missense rs2363956 G allele). For both C and D, confidence score (C-score) > − 1.5 indicates a model of correct global topology. The 3D structure follows rainbow coloring, where blue coloring represents the N-terminus, and red indicates the C-terminus. Kaplan Meier curves comparing *ANKLE1* gene expression and overall survival outcomes between low/medium and high *ANKLE1* expressing (**E**) EA and (**F**) AA, where high expression is shown in blue, and low/medium expression is shown in red. (**G**) KM curves comparing of overall survival between high expressing AA (blue) and high expressing EA (red). For (**E**–**G**), *N* values are reported for each comparison group, and the *p* value is reported on the plot.

In our TNBC risk analysis, we found that the minor G allele of the rs2363956 *ANKLE1* variant was protective against TNBC disease, which has previously been shown for ER-negative disease among AA^32^. Given its SIR-specific effect, we investigated the frequency of the allele across global 1000 genomes (1 KG) populations^33^. Population minor allele frequency (MAF) of the protective G allele is relatively equal among European and African groups (57% vs 50%, respectively, Table 1). However, among TNBC cases in our ICSBCS cohort, the frequency of the GG genotype is much lower in AA patients, compared to EA patients (14% and 43%, respectively) (Fig. 3B). This 20% drop in the minor allele frequency in TNBC cases among AA is what explains the interpreted potentially protective effect of the minor allele, inferring the major allele may somehow drive TNBC frequency higher in AAs (MAF_EA_ = 57.1%, MAF_AA_ = 37.2%).

To date, despite being repeatedly reported as a risk allele in both breast and ovarian cancer^32,34,35^, no investigation has linked a functional impact of this variant to risk or survival in this population. Given that the variant causes a dramatic amino acid change of leucine to tryptophan (L184W, Fig. 3A), there is a high probability that the protein structure is impacted, and subsequently have altered the function. We conducted a 3D rendering of the variant, comparing the structure of the protein with leucine at position 184 (Fig. 3C) to the minor allele change to tryptophan, and found a predicted destabilization of the gene product (Fig. 3D).

The allele’s protective effect through destabilization of ANKLE1 structure, together with its significant loss in AAs who suffer from higher rates of TNBC, suggests the major allele ANKLE1 protein could be a genetic driver of TNBC. We hypothesize that wildtype ANKLE1 expression suppresses TNBC progression, which is most frequently found in EA patients when caused by the rs2363956 variant. To further investigate this theory, we determined whether the expression of *ANKLE1* had any impact on survival^36^. We found that survival trends in TCGA breast cancer cases are significantly impacted by *ANKLE1* expression, but that the advantage of *ANKLE1* expression only benefits EA patients (Fig. 3E–G). Specifically, we found that when comparing high vs low/medium *ANKLE1* expression within SIR groups, EA have a significant survival improvement associated with higher expression (*p* = 0.035), but AA did not (*p* = 0.83) (Fig. 3E–F). In fact, when only including patients who had high expression of *ANKLE1*, EA had a longer survival advantage associated with *ANKLE1*, compared to AA (Fig. 3G, p = 0.052). This suggests that the benefit of *ANKLE1*, only found in EA, could be due to the 41–53% chance that EA are expressing the polymorphic version of *ANKLE1*, which harbors the rs2363956 allele.

## Discussion

While recent findings have delineated breast cancer risk alleles that pose increased or even decreased risk in African Americans specifically, many of these findings do not always replicate in other independent multi-ethnic cohorts. This is likely because of unmeasured individual admixture among the non-white individuals, who through social history are of mixed ancestry (i.e. Caribbean, Latin American and AAs) resulting from recent genetic admixture originating from multiple ancestor lineages^37–39^. This complexity of AA ancestry includes heterogeneity of African origins, spanning multiple African parental lineages through dozens of generations. This undoubtedly creates confounding genetic backgrounds that still pose a significant obstacle in identifying causal risk alleles among “African” Americans. However, measuring this genetic and ancestral diversity, and accounting for ancestry substructure would be a key first step toward clarifying the alleles that may be shared among individuals of common ancestry within SIR groups who display common disease/tumor types. Our latest race and West African ancestry adjustments in risk models demonstrate the power of combining diverse ancestral groups and utilizing ancestry estimates to clarify either false-positive or false-negative results if models do not properly consider the underlying ancestry/genetic background of the cohorts.

Our work represents a uniquely powered cohort that is enriched with a diverse cohort of patients and controls of African ancestry to directly investigate the impact of shared African ancestry in genetic risk for TNBC. We anticipate that our observations account for increased prevalence in women of African descent, at least in part. However, our analysis is still limited by the paucity of hormone receptor status in African cases and so the limited number of patients we can include in this analysis, thus far. Despite this limitation, we have robust findings that are compelling to expound upon in follow-up molecular and clinical studies.

First, our intention to replicate and verify the findings of AA-specific risk alleles is somewhat tenuous with associations fluctuating after adjustments for age and/or race. These covariate adjustments altering significance reflect the varying frequency of these alleles across these strata in our cohort and possibly more broadly in the population. Specifically, rs2981578, rs3745195 and rs4849887 were found to be significant prior to and after race adjustment, and lost significance with age adjustment, while rs2981579 and rs3112572 were found to be significant after race and age adjustment. For alleles that are in significantly different frequency across age categories, their distribution may reflect a difference in early vs. late onset cancers. For alleles that have significantly different frequency across race categories, their distribution may reflect ancestry-specific risk or population-private variants. Either scenario warrants a larger and more inclusive dataset to uncover genetic risk, robustly. This is an unmet need that could be essential to cancer prevention and much needed improvement for cancer risk prediction models.

We have validated our previous finding^31^ of the Duffy-null allele (rs2814778) as a TNBC-risk allele in our SIR all-inclusive analysis (OR = 3.814, *p* = 0.001). The Duffy-null allele is an ancestry-specific allele restricted to descendants of Sub-Saharan Africans. The allele arose among Sub-Saharan Africans and removed expression of DARC from erythrocytes, lending immunity from *Plasmodium vivax* malaria, as this malaria parasite utilized DARC as a portal of entry into erythrocytes^40,41^. The allele quickly swept to fixation across this population and is found at nearly ~ 100% among West Africans, and ~ 80% among AAs^42,43^. With the associations between WAa and TNBC that we and others have reported^31,44^, the potential association of the Duffy-null allele and TNBC is of great interest. With our expanded cohort analysis, we were able to perform the TNBC case-series risk assessment among SIR AAs only, and found that the risk was significantly retained among AA women after adjusting for both age and WAa (OR = 3.368, *p* = 0.007). This highlights that the Duffy null allele represents an ancestry-specific TNBC risk allele, and that the findings in our SIR all-inclusive analysis were not driven by ancestry-bias in our cohort. This is an important finding among our cohort, as the Duffy-null allele would not have been identified among previous GWAS studies underpowered with individuals of African ancestry.

Second, we have investigated the consequences of the protective rs2363956 variant on the *ANKLE1* gene coding region and uncovered a potential functional reason for race-group risk distinction. The allele has repeatedly been associated with breast and ovarian cancer risk and survival^34,35^, and this association has been replicated among AA women^32^. In the present analysis, we are the first to report that the ‘protective’ polymorphic ANKLE1 would be the more likely version expressed in EA patients, compared to AA or Ghanaian patients (GG genotype, 43%, 14% and 25%, respectively) (Fig. 3B). This suggests that the major T allele corresponds to a TNBC-specific oncogenic version of the *ANKLE1* gene. The potential mechanism of action for increased survival would appear to be DNA damage response, as ANKLE1 has repeatedly been shown to be involved in DNA repair pathways in pre-clinical and ex vivo screening, including endonuclease activity^45,46^, proliferation, and drug response hits in CRISPR screens in cancer cell lines^47–50^. Most intriguingly, one study in non-small-cell lung cancer indicated the combination of *ANKLE1* RNAi with paclitaxel increased the efficacy of the drug response^51^. Altogether, this is a very promising avenue for further investigation of targeted/combinatorial therapy, with potential to be transformative in treatment of TNBC, and with specific impact in AA who have higher expression of *ANKLE1*.

If validated through additional clinical studies, finding a novel oncogene specific to TNBC could be transformative in two ways: (i) to improve genetic risk models or create AA-tailored risk models, and (ii) to develop prognostic tests to inform survival prediction models, which currently do not include information about *ANKLE1*. Specifically, if we find that the patients who have longer survival carry the minor protective allele, correlated with higher expression of this polymorphic *ANKLE1*, we can quickly investigate if this is ultimately related to treatment response. Our preliminary data on survival trends certainly suggests this could be true.

The reported, albeit controversial, findings of TNBC mortality differences between women of African descent compared to women of European descent may be an important indicator of unknown differences in tumor biology. Here, we show that *ANKLE1* expression is linked to distinct survival outcomes, and this could potentially be linked to this polymorphic version of the *ANKLE1* gene. Intriguingly, this corresponds with differential impact of the gene’s expression on survival when comparing race groups among patients with high expression of the gene. While the functional consequence on mechanistic change is yet unknown, it is a clear indicator of survival and therefore a prognostic indicator. Excitingly, this also reveals a potential opportunity to develop immune-based inhibition of the oncogenic (major allele) version that is more likely expressed in AA. As the frequency of the oncogenic *ANKLE1* allele is higher in AA populations, this could present an opportunity for additional research to address its potential in precision therapies to bridge the survival gap in TNBC among race groups. Inclusion of diverse cohorts have powered this discovery and will drive clinical applications in the future.

## Methods

### International center for the study of breast cancer subtypes

The mission of the International Center for the Study of Breast Cancer Subtypes (ICSBCS) is to reduce the global breast cancer burden through advances in research and delivery of care to diverse populations worldwide. The ICSBCS brings together an international consortium of breast cancer clinicians and researchers, all of whom share the goal of addressing genetic and phenotypic variation in breast cancer risk and survival outcomes. We accrued prospective breast cancer patients from 2013 to 2017 as previously described^31^, extracting germline DNA from saliva samples collected at the time of consent at Komfo Anokye Teaching Hospital (KATH) in Kumasi, Ghana (N = 120), and St. Paul’s Millennium Hospital Medical College in Addis Ababa, Ethiopia. Additional cancer patient samples were collected at the Henry Ford Health System Hospital in Detroit, Michigan, and the University Cancer and Blood Center in Athens, GA (N_AA_ = 192 and N_EA_ = 184). The mean age is 47 ± 15.4 (mean ± sd) for Ghanaian patients, 59 ± 12.8 for AA and 60 ± 12.1 for EA. Healthy controls (N = 271) were recruited to the ICSBCS biospecimen registry through various sources of community engagement efforts throughout the US^52^ and the breast cancer screening clinic at KATH^22^. Informed consent was obtained from all individuals participating in the study, which was approved and under the regulation of the Weill Cornell Medical College (WCM) Institutional Review Board (IRB; protocol number 1807019405). All experiments were performed in accordance with the approved IRB protocol.

### Immunohistochemistry for BC tumor subtyping

For our TNBC case-series risk analysis, we determined hormone receptor status in our ICSBCS biospecimen registry via immunohistochemistry (IHC) methods that were described in detail in our previous study^31^. Expression of biomarkers was interpreted in accordance with the American Society of Clinical Oncology/College of American Pathologists guidelines^53,54^. Briefly, for estrogen and progesterone receptor IHC, staining of at least 1% was determined as positive. HER2/neu staining score of 0 or 1 + was determined as negative, and 3 + was determined as positive. HER2/neu staining score of 2 + was deemed equivocal and was further evaluated by fluorescent in situ hybridization. ICSBCS cases accrued in the USA were reviewed by the treating facility. IHC and pathology review of Ghanaian and Ethiopian cases was completed in Michigan (University of Michigan and Henry Ford Health System Hospital) and New York (Weill Cornell Medicine).

### Allele selection for BC case–control and TNBC case-series analyses

In our previous publication, we investigated nine reported AA BC risk variants in our African-enriched ICSBCS cohort, to determine BC or TNBC-specific risk within self-identified race (SIR) groups in our cohort. We additionally included the Duffy-Null allele (rs2814778), a promoter region variant of the *DARC/ACKR1* gene in our panel and demonstrated this allele to be a TNBC-specific risk allele among AA. Building upon our previous findings, we have both increased our number of samples across our SIR groups with genotypes available, and included an additional eight *DARC/ACKR1* gene variants in our panel that are implicated as ancestry-specific alleles, or sit in regions that are potentially involved in *DARC/ACKR1* gene regulation. These eight *DARC/ACKR1* gene variants represent upstream variants, 5′ UTR variants, and variants in the coding region of the gene. All alleles that were assessed in subsequent analyses are described in Table 1. Additionally, our African-enriched ICSBCS cohort allows us to also incorporate African ancestry measurements into the association model (below). PLINK (version 2.0)^55^ was used to assess linkage disequilibrium among these alleles, and no strong linkage disequilibrium was observed (maximum r^2^ of 0.44).

### Global ancestry estimation and genotyping of candidate alleles

Methods to determine global genetic ancestry have been previously reported in detail^31,56^. Briefly, DNA extracted from saliva samples were genotyped on the Sequenom MassARRAY iPLEX platform using an AIMs panel containing 100 markers specifically selected and validated for estimating continental ancestry among admixed populations^57,58^. The Sequenom TYPER software (version 4.0) was used for genotype calls, and STRUCTURE (version 2.3) was used to calculate admixture estimates for each individual^59^.

Similar to our global ancestry estimations, to obtain genotypes for our candidate variants for risk analyses (Table 1), DNA from saliva samples were genotyped for each of the variants using the Sequenom platform. For the Duffy-Null allele (rs2814778), we have obtained additional genotypes using single-target allele amplification reactions, as previously described^31^.

### Risk assessment

From our genotyping data, we used PLINK (version 2.0)^55^ to determine associations between the candidate variants and breast cancer risk in case–control analysis model, and TNBC-specific risk in case-series analysis model as previously described^31^. In both our BC and TNBC-specific risk analyses, we performed associations without covariates (non-adjusted), with SIR adjustment, and with SIR and age adjustments. We additionally investigated variant and risk associations within each SIR race group, where we performed analyses for non-adjusted and age-adjustments. For our analysis within SIR AA, using the genetic ancestry estimates, we were additionally able to adjust for West African ancestry in our models. For the candidate variants, we conducted the risk association using both a dominant and dosage statistical model^31^. In the dominance model where the genotypes are AA, Aa, aa (where a is minor allele), the resulting genotypes would be coded as 0, 1, 1 in the analysis model, where risk is weighted based on having at least one minor dominant allele. In the dosage model using the same genotypes, the resulting genotypes would be coded as 0, 1, 2, where the risk is weighted by the number of minor alleles present. In the main figures and tables, we show and discuss risk assessment output from the dosage models, where the full range of genotypes is considered in the analysis. In addition, the Benjamini–Hochberg method was used to adjust for multiple comparisons while controlling false discovery rate (FDR) at 0.05. FDR adjusted *p* values for Tables 2, 3, 4, and 5 are shown in Supplemental Tables 5–8, respectively.

For both the BC and TNBC-specific analyses, odds ratio output from the dosage risk assessment analyses were log transformed and plotted using the Forest Plot add-in (v8) within JMP Pro 15.0.0 statistical software (SAS Institute Inc., Cary, NC, 1989–2019).

### 3D modeling of ANKLE1 protein

We used the cBioPortal MutationMapper online program to visualize the *ANKLE1* protective variant rs2363956 in the context of the protein domain structure^60,61^. For 3D modeling of the wild type and rs2363956 missense variant, the ANKLE1 amino acid sequence in FASTA format was obtained from NCBI using the GrCh37.p13 reference and was submitted to I-TASSER^62–64^. The amino acid sequence is 615 residues long, and we performed 3D modeling to obtain the structure with and without the *ANKLE1* missense mutation included in our candidate variant analysis (rs2363956, L184W). The estimate of the accuracy of the predictions using I-TASSER is provided based on the confidence score (C-score) of the modeling. The C-score range is between [− 5, 2], where a C-score of a higher value suggests a model with higher confidence and vice-versa. Furthermore, Chimera program^65^ (version 1.14) was used for visualization and analysis of the predicted 3D ANKLE1 protein structure from I-TASSER.

### ANKLE1 survival analysis

The UALCAN online database was accessed to determine potential associations between gene expression and patient survival outcomes in the TCGA BC cohort^36^. *ANKLE1* gene expression was assessed across the patient cohort, and the upper quartile of expression was used to dichotomize expression into high and low/medium *ANKLE1* expressing individuals. The log rank *p* value obtained between comparison groups is reported on the plots.

## Supplementary Information

Supplementary Information.
