# Supplementary material for: Investigation of triple-negative breast cancer risk alleles in an International African-enriched cohort
Source: Sci Rep. 2021 Apr 29;11:9247. doi: 10.1038/s41598-021-88613-w (PMC8085076; doi:10.1038/s41598-021-88613-w)
Supplement: Supplementary file 1 — Supplementary Information. [file 41598_2021_88613_MOESM1_ESM.pdf]

TITLE

Investigation of Triple-Negative Breast Cancer Risk Alleles in an International African-enriched Cohort.

AUTHORS

Rachel Martini[1,2], Yalei Chen[3,4], Brittany D Jenkins[1,2], Isra A Elhussin[5], Esther Cheng[6], Syed A Hoda[6], Paula S Ginter[6], Jeffrey Hanover[7], Rozina B Zeidan[1], Joseph K Oppong[8], Ernest K Adjei[9], Aisha Jibril[10], Dhananjay Chitale[11], Jessica M Bensenhaver[12], Baffour Awuah[13], Mahteme Bekele[14], Engida Abebe[14], Ishmael Kyei[15], Frances S Aitpillah[8,15,], Michael O Adinku[15], Kwasi Ankomah[16], Ernest B Osei-Bonsu[13], Saul David Nathansan[12], LaToya Jackson[3], Evelyn Jiagge[3], Lindsay F Petersen[12], Erica Proctor[12], Petros Nikolinakos[17], Kofi K Gyan[1], Clayton Yates[5], Rick Kittles[18], Lisa A Newman[1] and Melissa B Davis[1*]

[1]Department of Surgery, Weill Cornell Medicine, New York, NY, USA; rnm4001@med.cornell.edu (RM), brittany.jenkins@nih.gov (BJ), roz4001@med.cornell.edu (RZ), kkg4001@med.cornell.edu (KG), lan4002@med.cornell.edu (LN), mbd4001@med.cornell.edu (MD)

[2]Department of Genetics, University of Georgia, Athens, GA, USA

[3]Department of Public Health Sciences, Henry Ford Health System, Detroit, MI, USA; ychen4@hfhs.org (YC), ljacks10@hfhs.org (LJ), ejiagge1@hfhs.org (EJ)

[4]Center for Bioinformatics, Henry Ford Health System, Detroit, MI, USA

[5]Department of Biology & Center for Cancer Research, Tuskegee University, Tuskegee, AL, USA; ielhussin0014@tuskegee.edu (IE), cyates@tuskegee.edu (CY)

[6]Department of Pathology and Laboratory Medicine, Weill Cornell Medicine, New York, NY, USA; esc9016@med.cornell.edu (EC), sahoda@med.cornell.edu (SH), psg9003@med.cornell.edu (PG)

[7]Rutgers New Jersey Medical School, Newark, NJ, USA; jth151@njms.rutgers.edu (JH)

[8]Department of Surgery, Komfo Anokye Teaching Hospital, Kumasi, Ghana; josephkoppong@yahoo.com (JO), fraitp@yahoo.com (FA)

[9]Department of Pathology, Komfo Anokye Teaching Hospital, Kumasi, Ghana; ernest24us@yahoo.com (EA)

[10]Department of Pathology, St. Paul's Hospital Millennium Medical College, Addis Ababa, Ethiopia; aisha.jibril@sphmmc.edu.et (AJ)

[11]Department of Pathology, Henry Ford Health System, Detroit, MI, USA; dchital1@hfhs.org (DC)

[12]Department of Surgery, Henry Ford Health System, Detroit, MI, USA; jbensen1@hfhs.org (JB), dnathan1@hfhs.org (SN), lpeters3@hfhs.org (LP), eprocto2@hfhs.org (EP)

[13]Directorate of Oncology, Komfo Anokye Teaching Hospital, Kumasi, Ghana; baff1470awuah@gmail.com (BA), ernest.bonsu@gmail.com (EB)

[14]Department of Surgery, St. Paul's Hospital Millennium Medical College, Addis Ababa, Ethiopia; mahteme.bekele@sphmmc.edu.et (MB), engida.abebe@sphmmc.edu.et (EA)

[15]Department of Surgery, Kwame Nkrumah University of Science and Technology, Kumasi, Ghana; raskyei@yahoo.com (IK), michaeladinku@gmail.com (MA)

[16]Directorate of Radiology, Komfo Anokye Teaching Hospital, Kumasi, Ghana; kyankomah@gmail.com (KA)

[17]University Cancer and Blood Center, Athens, GA, USA; pnikolinakos@universitycancer.com (PN)

[18]Department of Population Sciences, City of Hope Comprehensive Cancer Center, Duarte, CA, USA; rkittles@coh.org (RK)

*Corresponding Author

Melissa B Davis, PhD

Department of Surgery, Weill Cornell Medicine

420 E 70th Street, New York City, NY, USA 10021

Phone: (646) 962-2855

Email: mbd4001@med.cornell.edu

Supplemental tables 1-8

Supplemental Table 1. Breast cancer risk assessment (case-control) of previously identified variant alleles within SIR race groups

			SIR European Americans						***SIR African Americans***			***SIR Ghanaians***		
			Models without covariates			*Models with covariates**			*Models without covariates*			*Models without covariates*		
SNV ID	Associated/ Neighboring Genes	Minor Allele	*N*	OR (95% CI)	*P* value	*N*	OR (95% CI)	*P* value	*N*	OR (95% CI)	*P* value	*N*	OR (95% CI)	*P* value
rs13000023	*TNP1, DIRC3*	A	93	1.375 (0.334, 5.655)	0.659	92	1.415 (0.339, 5.908)	0.634	157	0.817 (0.238, 2.812)	0.749	139	1.104 (0.692, 1.760)	0.679
rs2363956	*ANKLE1*	G	96	1.504 (0.435, 5.200)	0.519	95	1.475 (0.417, 5.221)	0.547	154	1.363 (0.388, 4.790)	0.629	135	0.940 (0.591, 1.494)	0.793
**rs2981578**	***FGFR2***	T	91	1.101 (0.345, 3.517)	0.871	90	1.263 (0.378, 4.219)	0.704	152	1.990 (0.431, 9.183)	0.378	142	***2.030 (1.253, 3.289)***	***0.004***
rs2981579	*FGFR2*	G	94	0.861 (0.242, 3.066)	0.817	93	1.008 (0.272, 3.735)	0.990	152	1.281 (0.402, 4.087)	0.676	141	1.563 (0.970, 2.518)	0.067
rs3112572	*CASC16, LOC643714*	A	86	-		85	-		146	-		123	1.495 (0.863, 2.590)	0.152
**rs3745185**	***BABAM1***	A	90	0.978 (0.282, 3.397)	0.972	89	0.940 (0.250, 3.542)	0.927	151	0.394 (0.138, 1.127)	0.082	137	***0.545 (0.317, 0.936)***	***0.028***
rs4245739	*MDM4*	C	93	1.777 (0.418, 7.554)	0.436	92	1.889 (0.457, 7.803)	0.379	150	0.767 (0.232, 2.536)	0.663	139	0.855 (0.531, 1.377)	0.520
**rs4849887**	***LOC84934, GLI2***	C	89	0.450 (0.082, 2.476)	0.360	88	0.471 (0.079, 2.796)	0.407	152	1.382 (0.325, 5.880)	0.662	131	***2.472 (1.440, 4.243)***	***0.001***
**rs609275**	***MYEOV, CCND1***	T	89	-		88	-		149	***5.383 (1.015, 28.55)***	***0.048***	128	1.573 (0.870, 2.845)	0.134

***SIR models with covariates adjusts for age*

Supplemental Table 2. TNBC risk assessment (case-series) of previously identified variant alleles within SIR race groups

			SIR European Americans						***SIR African Americans***			***SIR Ghanaians***		
			Models without covariates			*Models with covariates**			*Models without covariates*			*Models without covariates*		
SNV ID	Associated/ Neighboring Genes	Minor Allele	*N*	OR (95% CI)	*P* value	*N*	OR (95% CI)	*P* value	*N*	OR (95% CI)	*P* value	*N*	OR (95% CI)	*P* value
rs13000023	*TNP1, DIRC3*	A	88	0.595 (0.148, 2.390)	0.464	87	0.590 (0.147, 2.369)	0.457	98	0.857 (0.449, 1.638)	0.641	11	0.133 (0.008, 2.181)	0.158
**rs2363956**	***ANKLE1***	G	91	1.272 (0.444, 3.644)	0.654	90	1.373 (0.470, 4.013)	0.563	97	***0.399 (0.222, 0.715)***	***0.002***	13	0.790 (0.170, 3.680)	0.766
rs2981578	*FGFR2*	T	86	1.659 (0.536, 5.134)	0.380	85	1.528 (0.483, 4.832)	0.470	93	1.165 (0.603, 2.251)	0.649	11	0.454 (0.038, 5.362)	0.530
rs2981579	*FGFR2*	G	89	1.954 (0.608, 6.276)	0.260	88	1.654 (0.501, 5.457)	0.409	93	0.890 (0.506, 1.563)	0.684	11	0.454 (0.038, 5.362)	0.530
rs3112572	*CASC16, LOC643714*	A	81	14.80 (0.801, 273.3)	0.070	80	11.70 (0.590, 232.2)	0.107	90	0.714 (0.372, 1.373)	0.313	10	-	0.988
rs3745185	*BABAM1*	A	85	1.097 (0.350, 3.434)	0.874	84	1.174 (0.359, 3.844)	0.790	93	0.556 (0.283, 1.090)	0.087	11	2.000 (0.125, 31.98)	0.624
rs4245739	*MDM4*	C	88	1.808 (0.679, 4.818)	0.236	87	1.621 (0.610, 4.308)	0.333	91	0.860 (0.453, 1.634)	0.645	11	0.750 (0.064, 8.834)	0.819
rs4849887	*LOC84934, GLI2*	C	84	2.062 (0.431, 9.880)	0.365	83	2.159 (0.420, 11.10)	0.357	94	0.479 (0.219, 1.045)	0.064	11	1.106 (0.140, 8.737)	0.924
rs609275	*MYEOV, CCND1*	T	84	1.646 (0.242, 11.20)	0.610	83	1.863 (0.233, 14.90)	0.558	92	1.173 (0.663, 2.077)	0.583	11	2.000 (0.125, 31.98)	0.624

**SIR models with covariates adjusts for age*

Supplemental Table 3. Breast cancer risk assessment (case-control) of *DARC/ACKR1* alleles within SIR race groups

			SIR European Americans						***SIR African Americans***			***SIR Ghanaians***		
			Models without covariates			*Models with covariates**			*Models without covariates*			*Models without covariates*		
SNV ID	Associated/ Neighboring Genes	Minor Allele	*N*	OR (95% CI)	*P* value	*N*	OR (95% CI)	*P* value	*N*	OR (95% CI)	*P* value	*N*	OR (95% CI)	*P* value
rs6676002	*DARC/ACKR1*	T	89	0.381 (0.097, 1.501)	0.168	88	0.350 (0.081, 1.511)	0.159	97	-	-	67	-	-
rs3027008	*DARC/ACKR1*	T	87	0.374 (0.097, 1.448)	0.155	86	0.334 (0.078, 1.422)	0.138	97	-	-	69	2.858 (0.394, 20.74)	0.299
**rs3027013**	***DARC/ACKR1***	T	87	***0.114 (0.017 ,0.757)***	***0.025***	86	***0.131 (0.019, 0.902)***	***0.040***	98	-	-	69	-	-
rs71782098	*DARC/ACKR1*	DEL	91	-	-	90	-	-	98	0.300 (0.049, 1.820)	0.191	72	1.423 (0.520, 3.892)	0.492
rs2814778	*DARC/ACKR1*	C	293	1.222 (0.110 ,13.64)	0.871	285	0.938 (0.076, 11.53)	0.960	223	1.000 (0.558, 1.794)	0.999	141	0.625 (0.074, 5.252)	0.665
rs17838198	*DARC/ACKR1*	T	90	3.763 (0.434 ,32.65)	0.229	89	5.671 (0.601, 53.48)	0.129	99	0.741 (0.080, 6.896)	0.792	80	-	-
rs3027016	*DARC/ACKR1*	G	87	1.873 (0.211 ,16.64)	0.573	86	1.72 (0.186, 15.94)	0.633	98	-	-	67	-	-
rs12075	*DARC/ACKR1*	G	89	2.961 (0.574 ,15.29)	0.195	88	4.103 (0.690, 24.42)	0.121	99	1.410 (0.170, 11.71)	0.750	74	-	-

***SIR models with covariates adjusts for age*

Supplemental Table 4. TNBC risk assessment (case-series) of *DARC/ACKR1* alleles within SIR race groups

			SIR European Americans						***SIR African Americans***			***SIR Ghanaians***		
			Models without covariates			*Models with covariates**			*Models without covariates*			*Models without covariates*		
SNV ID	Associated/ Neighboring Genes	Minor Allele	*N*	OR (95% CI)	*P* value	*N*	OR (95% CI)	*P* value	*N*	OR (95% CI)	*P* value	*N*	OR (95% CI)	*P* value
rs6676002	*DARC/ACKR1*	T	84	1.039 (0.2782, 3.879)	0.955	83	1.109 (0.281, 4.383)	0.883	90	-		2	-	-
rs3027008	*DARC/ACKR1*	T	82	0.790 (0.170, 3.665)	0.763	81	0.834 (0.175, 3.977)	0.820	90	-		2	-	-
rs3027013	*DARC/ACKR1*	T	82	-	-	81	-	-	90	-		2	-	-
rs71782098	*DARC/ACKR1*	DEL	86	4.222 (0.379, 47.06)	0.242	85	3.432 (0.269, 43.81)	0.343	90	2.316 (0.627, 8.556)	0.208	2	-	-
**rs2814778**	***DARC/ACKR1***	C	178	-	-	177	-	-	112	***3.871 (1.657, 9.046)***	***0.002***	31	2E06 (0, -)	0.977
rs17838198	*DARC/ACKR1*	T	85	1.459 (0.373, 5.709)	0.588	84	1.190 (0.278, 5.100)	0.815	91	0.632 (0.171, 2.328)	0.490	2	-	-
rs3027016	*DARC/ACKR1*	G	82	1.018 (0.191, 5.418)	0.983	81	1.083 (0.196, 5.977)	0.927	90	0.695 (0.156, 3.100)	0.633	2	-	-
rs12075	*DARC/ACKR1*	G	84	1.06 (0.304, 3.690)	0.927	83	0.949 (0.251, 3.588)	0.939	91	0.752 (0.290, 1.951)	0.558	2	-	-

**SIR models with covariates adjusts for age*

Supplemental Table 5. Breast cancer risk assessment (case-control) of previously identified variant alleles with FDR adjusted *p* values

			Overall BC risk (all samples)						***SIR African Americans***			***SIR Ghanaians***		
			Models without covariates			*Models with covariates**			*Models with covariates***			*Models with covariates***		
SNV ID	Associated/ Neighboring Genes	Minor Allele	*N*	OR (95% CI)	*FDR P* value	*N*	OR (95% CI)	*FDR P* value	*N*	OR (95% CI)	*FDR P* value	*N*	OR (95% CI)	*FDR P* value
rs13000023	*TNP1, DIRC3*	A	420	1.048 (0.756, 1.449)	0.823	271	1.061 (0.601, 1.874)	0.950	104	-	-	75	0.909 (0.466, 1.770)	0.849
rs2363956	*ANKLE1*	G	415	0.847 (0.629, 1.140)	0.450	274	1.272 (0.718, 2.254)	0.863	103	0.916 (0.135, 6.197)	0.975	76	1.409 (0.688, 2.890)	0.849
**rs2981578**	***FGFR2***	T	416	***1.508 (1.111, 2.047)***	***0.019***	267	1.036 (0.611, 1.759)	0.951	99	-	-	78	0.891 (0.481, 1.650)	0.849
rs2981579	*FGFR2*	G	418	1.246 (0.928, 1.675)	0.272	269	1.899 (1.063, 3.393)	0.264	99	-	-	77	1.792 (0.905, 3.545)	0.570
rs3112572	*CASC16, LOC643714*	A	385	1.007 (0.691, 1.467)	0.972	246	2.410 (1.086, 5.347)	0.264	96	-	-	65	2.088 (0.948, 4.597)	0.570
**rs3745185**	***BABAM1***	A	409	***0.666 (0.492, 0.901)***	***0.019***	265	0.618 (0.341, 1.119)	0.466	99	0.787 (0.104, 5.947)	0.975	77	0.590 (0.272, 1.280)	0.728
rs4245739	*MDM4*	C	409	1.193 (0.860, 1.654)	0.450	264	0.995 (0.569, 1.740)	0.986	97	0.008 (0.000, 20.16)	0.907	75	0.838 (0.427, 1.644)	0.849
**rs4849887**	***LOC84934, GLI2***	C	403	***1.654 (1.154, 2.371)***	***0.019***	257	0.790 (0.351, 1.776)	0.863	100	0.399 (0.013, 12.43)	0.975	69	0.754 (0.274, 2.073)	0.849
rs609275	*MYEOV, CCND1*	T	396	1.129 (0.822, 1.552)	0.592	253	1.121 (0.532, 2.361)	0.950	98	0.224 (0.014, 3.646)	0.907	67	0.920 (0.389, 2.176)	0.849

**Overall analysis models with covariates adjusts for age and SIR*

***SIR models with covariates adjusts for age*

Supplemental Table 6. TNBC-specific risk assessment (case-series) of previously identified variant alleles with FDR adjusted *p* values

			Overall TNBC risk (all samples)						***SIR African Americans***			***SIR Ghanaians***		
			Models without covariates			*Models with covariates**			*Models with covariates***			*Models with covariates****		
SNV ID	Associated/ Neighboring Genes	Minor Allele	*N*	OR (95% CI)	*FDR P* value	*N*	OR (95% CI)	*FDR P* value	*N*	OR (95% CI)	*FDR P* value	*N*	OR (95% CI)	*FDR P* value
rs13000023	*TNP1, DIRC3*	A	197	0.682 (0.410, 1.133)	0.159	190	0.781 (0.439, 1.387)	0.636	96	1.109 (0.538, 2.286)	0.881	6	-	-
**rs2363956**	***ANKLE1***	G	201	***0.593 (0.389, 0.903)***	***0.027***	194	***0.542 (0.332, 0.883)***	***0.112***	95	***0.420 (0.230, 0.769)***	***0.049***	8	1.471 (0.057, 38.26)	0.993
rs2981578	*FGFR2*	T	190	0.667 (0.435, 1.022)	0.087	183	1.248 (0.718, 2.169)	0.636	92	1.304 (0.656, 2.590)	0.881	6	17.55 (0.010, 29760)	0.993
rs2981579	*FGFR2*	G	193	0.790 (0.515, 1.212)	0.299	186	0.978 (0.599, 1.597)	0.930	92	0.942 (0.530, 1.672)	0.881	6	6.261E-08 (0, -)	0.993
rs3112572	*CASC16, LOC643714*	A	181	1.546 (0.880, 2.716)	0.159	174	0.748 (0.394, 1.421)	0.636	89	0.714 (0.367, 1.390)	0.751	5	6.976E-49 (0, -)	0.993
**rs3745185**	***BABAM1***	A	189	***0.503 (0.306, 0.843)***	***0.021***	182	0.682 (0.386, 1.203)	0.480	92	0.584 (0.288, 1.182)	0.479	6	3.304 (0.090, 120.90)	0.993
rs4245739	*MDM4*	C	190	0.906 (0.571, 1.438)	0.675	183	1.130 (0.658, 1.941)	0.820	90	0.861 (0.440, 1.685)	0.881	6	0.356 (0.004, 34.530)	0.993
**rs4849887**	***LOC84934, GLI2***	C	189	***0.414 (0.232, 0.738)***	***0.012***	182	0.666 (0.338, 1.313)	0.549	93	0.542 (0.241, 1.221)	0.479	6	0.676 (0.0470, 9.710)	0.993
**rs609275**	***MYEOV, CCND1***	T	187	***2.479 (1.593, 3.857)***	***<0.001***	180	1.245 (0.717, 2.163)	0.636	91	1.121 (0.610, 2.061)	0.881	6	14.23 (0.0261, 7750)	0.993

**Overall analysis models with covariates adjusts for age and SIR*
***SIR AA models with covariates adjusts for age and West African ancestry*
****SIR Ghanaian models with covariates adjusts for age*

Supplemental Table 7. Breast cancer risk assessment (case-control) of *DARC/ACKR1* alleles with FDR adjusted *p* values

			Overall BC risk (all samples)						***SIR African Americans***			***SIR Ghanaians***		
			Models without covariates			*Models with covariates**			*Models with covariates***			*Models with covariates***		
SNV ID	Associated/ Neighboring Genes	Minor Allele	*N*	OR (95% CI)	*FDR* *P* value	*N*	OR (95% CI)	*FDR* *P* value	*N*	OR (95% CI)	*FDR* *P* value	*N*	OR (95% CI)	*FDR* *P* value
rs6676002	*DARC/ACKR1*	T	282	1.290 (0.702, 2.368)	0.584	231	0.634 (0.164, 2.454)	0.863	96	-	-	47	-	-
rs3027008	*DARC/ACKR1*	T	282	1.187 (0.661, 2.131)	0.687	231	0.737 (0.230, 2.369)	0.863	96	-	-	49	2.270 (0.277, 18.60)	0.849
rs3027013	*DARC/ACKR1*	T	283	0.869 (0.372, 2.033)	0.823	231	0.250 (0.050, 1.241)	0.466	96	-	-	49	-	-
rs71782098	*DARC/ACKR1*	DEL	291	0.575 (0.307, 1.077)	0.178	238	0.753 (0.280, 2.028)	0.863	96	-	-	52	0.882 (0.298, 2.608)	0.849
**rs2814778**	***DARC/ACKR1***	C	712	***1.512 (1.263, 1.809)***	***< 0.001***	492	0.772 (0.392, 1.520)	0.863	153	0.696 (0.330, 1.467)	0.906	54	3.657 (0.231, 57.81)	0.849
**rs17838198**	***DARC/ACKR1***	T	299	***4.798 (2.125, 10.83)***	***< 0.001***	244	3.413 (0.678, 17.20)	0.466	97	1.052 (0.044, 25.41)	0.975	58	-	-
**rs3027016**	***DARC/ACKR1***	G	281	***4.586 (1.587, 13.26)***	***0.019***	229	2.311 (0.269, 19.88)	0.863	96	-	-	47	-	-
**rs12075**	***DARC/ACKR1***	G	292	***2.534 (1.498, 4.287)***	***< 0.001***	238	1.131 (0.382, 3.351)	0.950	97	1.108 (0.048, 25.80)	0.975	53	-	-

**Overall analysis models with covariates adjusts for age and SIR*

***SIR models with covariates adjusts for age*

Supplemental Table 8. TNBC-specific risk assessment (case-series) of *DARC/ACKR1* alleles with FDR adjusted *p* values

			Overall TNBC risk (all samples)						***SIR African Americans***			***SIR Ghanaians***		
			Models without covariates			*Models with covariates**			*Models with covariates***			*Models with covariates****		
SNV ID	Associated/ Neighboring Genes	Minor Allele	*N*	OR (95% CI)	*FDR* *P* value	*N*	OR (95% CI)	*FDR* *P* value	*N*	OR (95% CI)	*FDR* *P* value	*N*	OR (95% CI)	*FDR* *P* value
**rs6676002**	***DARC/ACKR1***	T	176	***0.191 (0.058, 0.635)***	***0.019***	175	0.403 (0.111, 1.460)	0.480	90	-	-	2	-	-
**rs3027008**	***DARC/ACKR1***	T	174	***0.134 (0.032, 0.568)***	***0.019***	173	0.275 (0.060, 1.261)	0.448	90	-	-	2	-	-
rs3027013	*DARC/ACKR1*	T	174	-	-	173	-	-	90	-	-	2	-	-
**rs71782098**	***DARC/ACKR1***	DEL	178	***3.403 (1.231, 9.412)***	***0.029***	177	2.629 (0.796, 8.682)	0.448	90	2.547 (0.668, 9.716)	0.479	2	-	-
**rs2814778**	***DARC/ACKR1***	C	339										-	-
**rs17838198**	***DARC/ACKR1***	T	178	***0.367 (0.164, 0.821)***	***0.027***	177	0.929 (0.355, 2.430)	0.930	91	0.722 (0.190, 2.754)	0.881	2	-	-
rs3027016	*DARC/ACKR1*	G	174	0.390 (0.144, 1.058)	0.087	173	0.839 (0.270, 2.609)	0.871	90	1.204 (0.226, 6.422)	0.881	2	-	-
**rs12075**	***DARC/ACKR1***	G	177	***0.380 (0.199, 0.726)***	***0.012***	176	0.846 (0.396, 1.807)	0.820	91	0.922 (0.319, 2.669)	0.881	2	-	-

**Overall analysis models with covariates adjusts for age and SIR*

***SIR AA models with covariates adjusts for age and West African ancestry*

****SIR Ghanaian models with covariates adjusts for age*
